# Excessive Heat and Respiratory Hospitalizations in New York State: Estimating Current and Future Public Health Burden Related to Climate Change

**DOI:** 10.1289/ehp.1104728

**Published:** 2012-08-24

**Authors:** Shao Lin, Wan-Hsiang Hsu, Alissa R. Van Zutphen, Shubhayu Saha, George Luber, Syni-An Hwang

**Affiliations:** 1 Center for Environmental Health, New York State Department of Health, Albany, New York, USA; 2 Department of Epidemiology and Biostatistics, University at Albany School of Public Health, Rensselaer, New York, USA; 3 National Center for Environmental Health, Centers for Disease Control and Prevention, Atlanta, Georgia, USA

**Keywords:** climate change, extreme heat, morbidity, projection, public health burden, respiratory disease

## Abstract

Background: Although many climate-sensitive environmental exposures are related to mortality and morbidity, there is a paucity of estimates of the public health burden attributable to climate change.

Objective: We estimated the excess current and future public health impacts related to respiratory hospitalizations attributable to extreme heat in summer in New York State (NYS) overall, its geographic regions, and across different demographic strata.

Methods: On the basis of threshold temperature and percent risk changes identified from our study in NYS, we estimated recent and future attributable risks related to extreme heat due to climate change using the global climate model with various climate scenarios. We estimated effects of extreme high apparent temperature in summer on respiratory admissions, days hospitalized, direct hospitalization costs, and lost productivity from days hospitalized after adjusting for inflation.

Results: The estimated respiratory disease burden attributable to extreme heat at baseline (1991–2004) in NYS was 100 hospital admissions, US$644,069 in direct hospitalization costs, and 616 days of hospitalization per year. Projections for 2080–2099 based on three different climate scenarios ranged from 206–607 excess hospital admissions, US$26–$76 million in hospitalization costs, and 1,299–3,744 days of hospitalization per year. Estimated impacts varied by geographic region and population demographics.

Conclusions: We estimated that excess respiratory admissions in NYS due to excessive heat would be 2 to 6 times higher in 2080–2099 than in 1991–2004. When combined with other heat-associated diseases and mortality, the potential public health burden associated with global warming could be substantial.

The global average surface temperature is likely to rise and heat waves will be more intense and frequent in the future warmer climate [Intergovernmental Panel on Climate Change (IPCC) 2007]. Although several studies have projected heat-related mortality (Bambrick et al. 2008; Campbell-Lendrum and Woodruff 2007; Knowlton et al. 2007), few have evaluated the public health burden associated with morbidity (Bambrick et al. 2008). Previous studies have suggested substantial increases in mortality as a result of extreme heat. For example, Knowlton et al. (2007) projected New York City regional increases in heat-related premature mortality by the 2050s ranging from 47% to 95%, with a mean 70% increase compared with the 1990s. Most studies have reported a strong relationship between extreme heat days and respiratory admissions. For each 1°C increase in maximum apparent temperature (AT) above a threshold, Michelozzi et al. (2009) estimated a 4.5% increase in respiratory admissions for the ≥ 75-year age group in Mediterranean cities. Lin et al. (2009) reported that each 1°C increase above the threshold of the temperature–health effect curve in different regions of New York City [29–36°C (84.2–96.8°F)] was associated with a 2.7–3.1% increase in same-day respiratory admissions there. However, few of these studies projected the public health burden attributable to extreme heat, including attributable risk, cost, and loss of productivity. Because this information is important for public health preparedness and responses, we sought to assess the excess current and future public health impacts of respiratory disease attributable to extreme heat in summer, including the number of admissions, hospitalization costs, days hospitalized, and lost productivity from days hospitalized across multiple regions of New York State (NYS).

## Materials and Methods

*Data sources*. We obtained respiratory hospitalization data from 1991–2004 among NYS residents from the NYS Department of Health Statewide Planning and Research Cooperative System (SPARCS), a legislatively mandated database of hospital discharge data for approximately 95% of all NYS acute care admissions, excluding admissions to psychiatric and federal hospitals (NYS Department of Health 2002). Data included principal diagnosis, admission and discharge dates, sources of payment, total charges, date of birth, sex, race, ethnicity, and residential street address. About 94% of addresses were geocoded to street level and 5% to ZIP code level. Less than 1% of addresses could not be geocoded and were excluded from the analysis. Family income information was obtained from 1990 and 2000 U.S. Census data at the census block level (U.S. Census Bureau 1992, 2002).

Meteorological data for NYS during 1991–2004, including hourly observations for temperature, barometric pressure, and dew point, were provided by the Data Support Section of the Computational and Information Systems Laboratory at the National Center for Atmospheric Research (NCAR; Boulder, CO) from airport weather stations. Hourly ambient ozone data were obtained from the NYS Department of Environmental Conservation (Albany, NY) ambient air monitors. We used an 8-hr maximum average ozone concentration limited to the 1000–1800 hours, which represents the most likely time for outdoor exposure. Both meteorological and ambient air monitoring locations were geocoded to street level. Projections of the extent and geographical climate distribution were obtained from the IPCC (NCAR 2007a, 2007b, 2009), including projected temperature, barometric pressure, and specific humidity data for 2046–2065 and 2080–2099 (i.e., about 50 and 100 years from the baseline period).

*Study population and health outcomes.* The study population included all NYS residents. We assessed respiratory admissions, related hospitalization costs, days hospitalized, and lost productivity from days hospitalized. Specifically, we counted all hospital admissions in the summer (June–August) with a principal diagnosis of respiratory disease among NYS residents during 1991–2004. Based on the *International Classification of Disease, 9th Revision, Clinical Modification* (ICD-9; Department of Health and Human Services 1997), respiratory diseases included chronic bronchitis (ICD-9 code 491), emphysema (ICD-9 code 492), asthma (ICD-9 code 493), and chronic obstructive pulmonary disease (COPD; ICD-9 code 496). For children ≤ 4 years of age, we included acute bronchitis and bronchiolitis (ICD-9 code 466) and bronchitis, not specified as acute or chronic (ICD-9 code 490) because these respiratory illnesses are difficult to distinguish from asthma among very young children. The hospitalization charges listed in SPARCS do not reflect actual inpatient hospitalization costs; therefore, we multiplied the hospitalization charges indicated in SPARCS by the average cost-to-charge ratio (0.54) for NYS obtained from the Healthcare Cost and Utilization Project (2011). We used the length of stay for each patient to estimate the economic cost of lost productivity from days hospitalized according to market and household productivity estimates for U.S. adults by age and sex (Grosse et al. 2009).

*Meteorological and exposure indicators*. We identified 14 weather regions with relatively homogeneous weather and ozone exposures by overlaying and merging the 10 NYS climate divisions (National Climate Data Center, Asheville, NC) with the 11 ozone regions developed for NYS by Chinery and Walker (2009). Each hospitalization was assigned to a weather region on the basis of geocoded residential address.

Daily mean apparent temperature (AT; an index of human discomfort resulting from the combined effects of heat and humidity) was calculated in degrees Celsius as AT = –2.653 + 0.994T + [0.0153 × (dewpoint)^2^] as described previously (Kalkstein and Valimont 1986, Steadman 1979), where T represents daily mean temperature. Although the relationship between temperature or AT and respiratory disease is usually U- or V-shaped (Linares and Diaz 2008; McMichael et al. 2008), we used a linear-threshold model to quantify the effect of high temperature. The threshold (T_0_) was selected for each region after modeling all possible values (70–105°F) and selecting the one with the lowest model deviance for each region (Armstrong 2006; McMichael et al. 2008). We also identified two alternate extreme heat indicators: *a*) the 90th percentile of AT based on the summer AT distribution from 1991–2004, and *b*) daily AT > 90°F.

*Climate scenarios*. The IPCC has defined a range of possible future trends in greenhouse gas emissions (IPCC 2007). The scenarios presented in the IPCC *Special Report on Emissions Scenarios* (SRES) (IPCC 2000) are plausible indications of what the future could be like over decades or centuries (IPCC-Task Group on Data and Scenario Support for Impacts and Climate Analysis A 2007). To represent a wide range of possible future climates, we selected three of the SRES scenarios—high (A2), mid (A1B), and low (B1) emissions—based on alternative assumptions about changes in economy, technology, demographics, and energy demand (IPCC 2000, 2007). A2 assumes a very heterogeneous world with continuously increasing population growth, slow and regionally oriented economic development, and slow technological change. A1B assumes a world of very rapid economic growth, a global population that peaks in midcentury and then gradually declines, and rapid introduction of new and more efficient technologies with a balance across all energy sources. B1 assumes a convergent world, with the same population growth as in scenario A1B, but with more rapid changes toward a service and information economy and with a reduction in material intensity and clean and resource-efficient technologies (IPCC 2000, 2007).

*Projection of future summer AT distributions*. We estimated future AT by using temperature, barometric pressure, and specific humidity obtained from the IPCC (NCAR 2007a, 2007b, 2009), which applied the Community Climate System model Version 3 (CCSM3), based on the three climate scenarios described above and constructed according to longitude, latitude, and time with grid cells of 155 km × 155 km. We assumed that regional variation in climate across the 14 weather regions at baseline would remain unchanged. We used the change in spatially averaged mean summer daily AT for each region from baseline to midcentury (2046–2065) and the end of the century (2080–2099) under each climate scenario (Bambrick et al. 2008; McMichael et al. 2004).

*Statistical analysis*. To assess public health impacts, we estimated the relationship between daily temperature variation and respiratory admissions using a two-stage Bayesian model that included a regional analysis and a statewide estimate adjusted for regional confounders. In stage 1, we estimated the association between extreme heat and respiratory disease hospitalization for each of the 14 NYS regions using generalized additive models (GAM) (Hastie and Tibshirani 1989) with Poisson distributional errors and a log link function in SAS (version 9.2; SAS Institute Inc., Cary, NC). We assumed a log-linear increase in health risk above a temperature threshold (T_0_), which was determined by comparing the maximum likelihood estimates over all possible threshold values in the range of data and using the value with the lowest deviance. We used a linear association between hospitalization and each 1°F increase in AT > T_0_ to estimate the extreme heat effect for each region:

log(count) = α_0_ + s(AT < T_0_, df) + β_0_(AT > T_0_) + s(date, df) + s(O_3_, df) + β_1_ +…+ β_8_ + ε, [1]

where T_0_ is the threshold value of AT, β_0_ is the slope parameter for AT > T_0_ (representing the risk of hospitalization with each 1°F increase in AT > T_0_), and α_0_ and ε are the intercept and error terms, respectively. Spline curves, indicated by s(AT < T_0_, df), s(date, df), and s(O_3_, df), were used to model the effects of AT < T_0_, long-term trends and seasonal variation (date), and ozone (O_3_). Degrees of freedom (df) were determined using an automatic procedure based on minimizing the sum of absolute values for the first 30 items of the partial autocorrelation function of the model residuals (Armstrong 2006). We also controlled the effect of day of the week (with Monday–Saturday represented by β_1_–β_6_), and the Northeast blackout events that occurred on 14 and 15 August 2003 (β_7_, β_8_). Model fit was assessed by Bartlett’s Kolmogorov–Smirnov statistic (Bartlett 1978). We also checked the model residuals for autocorrelation and partial autocorrelation functions to rule out seasonality or other patterns (Armstrong 2006; Lin et al. 2009).

In stage 2, we pooled region-specific estimates to generate a statewide estimate using a Bayesian hierarchical model (Dominici et al. 2002). We controlled for region-level covariates using yearly data estimated from 1990 and 2000 U.S. Census data, including population density, health-care access (minimum distance to clinics), race and ethnicity (percent of black and Hispanic residents), percent of residents with ≤ high school education, mean apparent temperature during June–August, percent living below the poverty level, and percent of the regional population that were elderly (age ≥ 75 years) and living alone. Pooling of information across regions can potentially improve statistical power and the generalizability of the results, as well as account for geographic heterogeneity of effects (Dominici 2002). All second stage analyses were conducted using tlnise in R, version 0.2-7 (Everson and Morris 2000).

Daily excess admissions attributable to extreme heat were computed at baseline (1991–2004) and projected for 2046–2065 and 2080–2009 as

*A* = *R* × Δ*T* × *M*, [2]

where *A* represents the estimated number of daily admissions attributable to AT > T_0_ for each time period; *R* is the estimated percentage increase in admissions per 1°F increase in AT > T_0_ at baseline based on Equation 1 [i.e., *R* = exp(β_0_) – 1]; *M* is the mean number of daily respiratory admissions during June–August at baseline; and ΔT is the difference between the mean daily AT for each time period and the baseline T_0_ on days when AT > T_0_. We used the thresholds identified from the baseline data (1991–2004) for each region to project future impacts.

Daily excess hospitalization costs and days of hospitalization attributable to extreme heat were computed as

*C* = *A* × *D*, [3]

where *C* represents either daily temperature-attributable hospitalization costs or days hospitalized; *A* is as defined in Equation 2; and *D* is the average number of days hospitalized per hospitalization at baseline (Campbell-Lendrum and Woodruff 2006, 2007; McMichael et al. 2004). The average cost per day of hospitalization was adjusted for inflation (by month and year) for each time period and standardized to 2004 U.S. dollars to ensure that costs were comparable across the different years in our study (InflationData.com 2011). Because a dollar in the future is considered to be of less value than a current dollar, it is common practice in health cost estimation to discount for future costs incurred across different time periods to derive the current worth of all future amounts. We used an annual discount rate of 3% as recommended by the U.S. Panel on Cost-Effectiveness in Health and Medicine to estimate the 2004 dollar value of the future stream of costs (Goodman 2004; Phillips and Chen 2002). Excess lost productivity from days hospitalized was computed by multiplying estimated excess days of hospitalization by age-specific daily production values presented by Grosse et al. (2009).

Stratified analyses were conducted based on individual level data such as sex, age, specific disease group, insurance type, and census-block group–level family income. We also conducted a sensitivity analysis using another global climate model, the Centre National de Recherches Meteorologiques Coupled Global Climate Model Version 3 (CM3) (Salas 2005a, 2005b, 2005c), to compare and validate our results. Moreover, we performed sensitivity analyses to examine whether the estimate of excess heat-related health burden was due to population composition changes in age distribution or race/ethnicity. We used population growth percentages on these specific subgroups to roughly estimate excess admissions from the subgroups without population growth and those with population growth according to U.S. population projections from 1998–2000, 2050–2070, and 2075–2100 (U.S. Census Bureau 2000) and the U.S. Hispanic population from 2000 to 2050 (U.S. Census Bureau 2011a).

## Results

*Regional analysis*. During the baseline period, the LaGuardia Airport (LGA) region had the largest estimated increases in excess admissions (32/year) followed by the Great Lakes–Rochester (10/year), White Plains (8/year), and John F. Kennedy Airport (JFK; 8/year) regions (Table 1). The LGA region also had the highest estimated excess hospitalization costs (US$226,228) and days spent in the hospital (179). Estimated health impacts were also highest for the LGA region when projected for 2046–2065 and 2080–2099 under the mid-emissions scenario (A1B; 65 and 92 hospital admissions, respectively). However, the Binghamton (35 and 49 admissions), Long Island (26 and 36 admissions), and JFK (25 and 35 admissions) regions ranked 2nd, 3rd, and 4th for both time periods.

**Table 1:** Baseline and projected changes in respiratory admissions, hospitalization costs (US$), and days hospitalized associated with AT > T0 estimated for 14 NYS weather regions.

Baseline (1991–2004)							2046–2065^a^			2080–2099^a^		
Region	T_0_	Percent change in risk/°F > T_0_	Average cost per admission	Average days hospitalized per admission	Excess admissions per year^b^	Excess cost per year^c^	Excess days hospitalized per year^d^	Excess admissions per year^b^	Excess cost per year^e^	Excess days hospitalized per year^d^	Excess admissions per year^b^	Excess cost per year^f^	Excess days hospitalized per year^d^
LGA	86	0.81*	$7,013	5.54	32	$226,228	179	65	$2,006,135	362	92	$12,377,786	509
JFK	85	1.11	$7,364	6.04	8	$56,767	47	25	$811,150	152	35	$5,021,408	214
Staten Island	87	1.06	$6,554	5.83	2	$13,062	12	4	$121,283	25	6	$769,009	36
Long Island	85	0.93	$8,003	6.88	6	$46,459	40	26	$899,311	176	36	$5,465,560	244
White Plains	74	0.36	$7,543	7.59	8	$59,111	59	20	$645,145	148	23	$3,343,564	175
Hudson Valley South	81	2.30*	$7,390	6.59	6	$47,955	43	14	$455,001	93	20	$2,809,536	130
Hudson Valley North	86	–1.96	$4,574	6.23	–1	–$2,308	–3	–2	–$48,914	–15	–4	–$370,931	–26
Adirondack and North	78	–2.46	$3,792	5.84	–4	–$13,709	–21	–5	–$90,110	–32	–8	–$565,960	–45
Mohawk Valley	77	0.80	$4,561	6.21	2	$9,991	14	4	$75,957	24	5	$459,694	33
Binghamton	79	3.67*	$4,552	5.94	4	$18,016	24	35	$705,515	210	49	$4,292,689	291
Great Lakes–Rochester	74	1.35	$4,177	5.83	10	$43,139	60	18	$326,864	104	24	$1,911,475	139
Central Lakes	77	0.75	$4,245	6.03	4	$17,554	25	5	$91,129	30	7	$565,360	42
Western Plateau	71	0.62	$3,922	5.85	4	$14,832	22	10	$173,355	59	12	$940,607	73
Great Lakes– Buffalo	82	0.89	$4,640	6.19	2	$10,107	13	4	$86,286	26	7	$627,997	44
aProjections based on the CCSM3 climate model assuming SRES emissions scenario A1B. bCalculated using Equation 2. cAverage cost per admission × excess admissions. dAverage days hospitalized × excess admissions. eAverage cost per admission × excess admissions × [1 + discount rate (0.03)]50. fAverage cost per admission × excess admissions × [1 + discount rate (0.03)]100. *p < 0.05.																

*Statewide analysis*. We observed a statistically significant increase in percent change in risk per 1°F above threshold for 1991–2004 for NYS overall, with 99 excess hospital admissions, US$0.64 million in excess cost, and 616 days of excess hospitalization per year attributable to extreme heat (Table 2). The increase in hospital admissions attributable to heat among females was significantly greater than the estimated increase among males. In addition, we observed a significant increase in percent change in risk per 1°F among neighborhoods with a high percentage of people with low income. The lower income group also had a greater estimated increase in admissions than the higher income group but a smaller increase in hospitalization costs and days spent in the hospital. Within disease categories, the largest excess was in bronchitis admissions.

**Table 2:** Estimated respiratory hospital admissions, hospitalization costs (US$), and days hospitalized associated with AT > T0 at baseline (1991–2004) by demographic and disease subgroups in NYS.

Percent change in risk/°F > T_0_	Excess admissions per year	Excess cost per year	Excess days hospitalized per year
Sex
Female	1.35*	82	$555,717	533
Male	0.38	17	$106,743	102
Age (years)
0–15	–0.33	–6	–$20,088	–18
16–64	0.93	40	$239,494	219
65–74	1.16	24	$199,609	196
≥ 75	1.17	27	$236,496	238
Diseasea
Asthma	0.47	26	$129,927	117
Bronchitis	1.14	41	$300,782	288
Other	1.49	23	$227,016	242
Incomeb
Low	1.26*	68	$412,910	398
High	1.16	61	$423,279	404
Insurancec
No insurance	0.93	4	$14,697	13
Medicare	0.05	2	$18,048	18
Medicaid	–0.01	0	–$1,446	–1
Private insurance	0.64	19	$101,981	89
All	0.93*	99	$644,069	616
aDisease groups defined by ICD-9 codes in SPARCS: asthma, ICD-9 code 493; bronchitis, ICD-9 codes 491, 466 (for age < 5 years), and 490 (for age < 5 years); other, ICD-9 codes 492 and 496. bLow income ≤ median; high income > median. cDefined by sources of payment in SPARCS (NYS Department of Health 2002). *p < 0.05.								

Although 16- to 64-year-olds had the largest estimated admissions attributable to extreme heat and individuals ≥ 75 years of age had the largest excess days hospitalized, excess hospitalization costs were similar between the two age groups at baseline (Table 2). For days hospitalized, individuals in the ≥ 75, 65–74, and 55–64 year age groups ranked 1st, 2nd, and 3rd (Table 3). However, excess lost productivity from days hospitalized in summer was the largest within 55- to 64-year-olds (US$12,830), followed by the 65–74 and ≥ 75-year-olds.

**Table 3:** Estimated days hospitalized and lost productivity (US$) for respiratory admissions associated with AT > T0 at baseline (1991–2004) by age group in NYS.

Excess days hospitalized per year	Personal daily production value^a^	Excess lost productivity from days hospitalized per year^b^
Age
16–24	10	$55.64	$556
25–34	20	$149.13	$2,982
35–44	36	$176.47	$6,353
45–54	57	$172.29	$9,821
55–64	96	$133.65	$12,830
65–74	196	$64.73	$12,687
≥ 75	238	$42.57	$10,132
aData from Grosse et al. (2009). bExcess days hospitalized × personal daily production value.						

The mean summer AT during the baseline period in NYS was 72.13°F (Table 4). The high-emissions scenario (A2) had the highest projected AT, followed by the mid- (A1B) and low- (B1) emissions levels. For the low-emissions scenario (B1), the mean summer AT projected for 2080–2099 was slightly higher than the mean AT projected for 2046–2065 (75.59°F vs. 75.12°F). Relative to baseline, the projected summer mean AT for 2080–2099 increased 4.8% under the low-emissions scenario (B1), 8.2% under the mid-emissions scenario (A1B), and 14.8% under the high-emissions scenario (A2), and ranged from 3.46°F to 10.68°F. In 50 years, the estimated number of respiratory disease hospital admissions in NYS attributable to extreme heat ranged from 190 to 260 cases (1.9–2.6 times greater than baseline), resulting in US$5.5–$7.5 million in related hospitalization costs, 1,202–1,630 days of hospitalization, and US$0.47–$0.64 million in lost productivity from days hospitalized per year, compared with US$55,361 in lost productivity per year in 1991–2004. In 100 years, the estimated number of hospital admissions for respiratory disease in NYS attributable to extreme heat ranged from 206 to 607 (2.1–6.1 times greater than baseline), resulting in US$26–$76 million in related hospitalization costs, 1,299–3,744 days hospitalized, and US$2.2–$6.5 million in lost productivity from days hospitalized per year. For the low-emissions scenario, the estimated health impacts in 100 years are slightly higher than those in 50 years.

**Table 4:** Comparison of estimated statewide respiratory admissions, hospitalization costs (US$), days hospitalized, and lost productivity (US$) at baseline and projected using three emissions scenarios in NYS.

Time, scenario	Mean summer daily AT (°F)	Admissions (ratio)^a^	Cost of hospitalization^b^	Days hospitalized	Lost productivity from days hospitalized^c^
Baseline (1991–2004)	72.13	99	$644,069	616	$55,361
50 years (2046–2065) low	75.12	190 (1.9)	$5,497,603	1,202	$471,482
50 years (2046–2065) mid	76.19	236 (2.4)	$6,852,002	1,484	$582,746
50 years (2046–2065) high	76.97	260 (2.6)	$7,490,615	1,630	$639,865
100 years (2080–2099) low	75.59	206 (2.1)	$26,045,504	1,299	$2,234,027
100 years (2080–2099) mid	78.01	318 (3.2)	$40,429,610	1,988	$3,423,747
100 years (2080–2099) high	82.81	607 (6.1)	$76,334,071	3,744	$6,450,926
Scenarios: low, B1; mid, A1B; high, A2. aProjected number of admissions/baseline number of admissions (1991–2004). bStandardized to August 2004; baseline cost was adjusted for inflation rates, and current cost was adjusted to future values by an annual discount rate of 3%. cFuture cost estimates were adjusted for inflation and a discount rate of 3%.										

**Figure 1:**
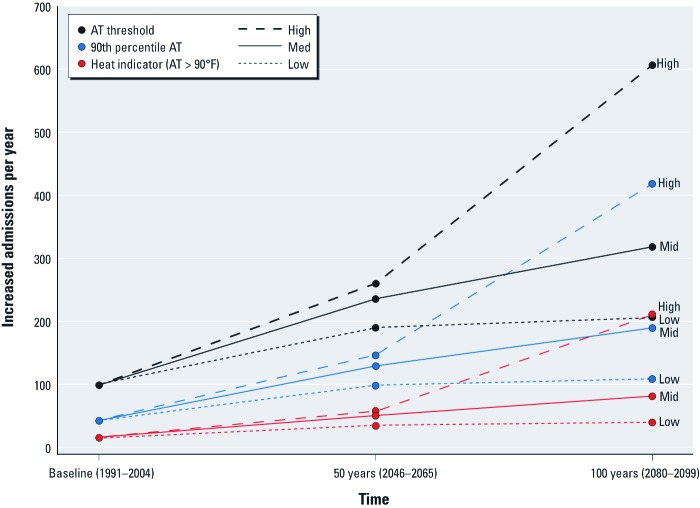
Projected statewide increased summer respiratory admissions per year under various times, scenarios, and threshold indices for NYS.

We also examined respiratory admissions per year under the three climate scenarios and two alternate heat indicators (Figure 1). For each heat indicator, the high-emissions scenario (A2) had the highest annual increase in admissions, followed by the mid- (A1B) and low-emissions (B1) scenarios. For each climate scenario, the estimated increase in hospital admissions was greatest for the default AT threshold, followed by the 90th percentile AT and the heat indicator (> 90°F AT).

To address uncertainty, we conducted sensitivity analyses to estimate potential excess heat-related health risks after accounting for projected increases in proportions of elderly and Hispanic people in the NYS population. After accounting for an aging population, the heat-attributable risks under the A1B scenario was 2.3 and 3.7 times greater than our original estimates for 2046–2065 and 2080–2099, respectively (data not shown). Similarly, the excess heat-related risk under the A1B scenario after incorporating the projected increase in Hispanics was 3.8 times the original estimate for 2046–2065 (data not shown). Estimates based on an alternate global climate model (CM3) in 2046–2065 and 2080–2099 were 1.5–2.0 times higher than our original estimates, depending on the scenario (data not shown).

## Discussion

According to our estimates, the proportional increase in respiratory admissions per year due to extreme heat in the summer would be 90–160% and 110–510% higher than baseline in 2046–2065 and 2080–2099, respectively. In terms of economic impact, we estimated that baseline hospitalization costs related to increased respiratory diseases due to high temperature in summer were US$0.64 million per year, and that projected costs would be US$5.5–$7.5 million and US$26–$76 million in 2046–2065 and 2080–2099, respectively. Differing results between our study and previous studies are difficult to interpret because of differences in the outcomes assessed. Bambrick et al. (2008) predicted that, relative to 1990, the total annual number of temperature-related hospital admissions in Australia would increase 185–186% by 2050 and 217–223% by 2100, which is lower than the 510% increase that we projected for respiratory disease admissions in NYS under the high-emissions scenario in 2080–2099. Peng et al. (2011) projected an excess of 166–2,217 deaths/year in Chicago in 2081–2100 attributable to heat waves using the A2, A1B, and B1 climate change scenarios. In a study based on climate scenarios provided by the UK Climate Impacts Program (UKCIP), Donaldson et al. (2001) estimated a 253% increase in annual heat-related mortality in the United Kingdom for the 2050s under the medium-high UKCIP scenario for temperature increase. Using a range of SRES scenarios (A1, A2, B1, B2), Dessai (2003) projected that annual heat-related death rates in Lisbon, Portugal, will increase from 5.4–6 per 100,000 for 1980–1998 to 7.3–35.6 per 100,000 by the 2050s.

In the present study, the projected future public health burden of respiratory admissions due to extreme heat varied greatly across the 14 NYS weather regions. We found that multiple regions, such as Binghamton and Hudson Valley South, would have larger proportional increases in respiratory admissions due to extreme heat than other NYS regions. This finding is consistent with the study by Knowlton et al. (2007), which projected that these counties including Dutchess (which overlaps with our Hudson Valley South region), Orange, Ulster (including parts of the Hudson Valley South and Binghamton regions), and Sullivan (in the Binghamton region), will experience larger proportional increases in heat-related mortality due to larger increases in mean daily temperatures by the 2050s. However, extreme heat will have larger absolute impacts in New York City and other urban areas that have larger populations and higher proportions of vulnerable populations. Knowlton et al. (2007) estimated a mean percentage increase in heat-related premature mortality of 38–72% in metropolitan New York City by 2050, which is consistent with our projection for respiratory admissions in the LGA region, which includes most of New York City and had the highest estimated number of admissions and related burdens in all three time periods.

We found significant health risks and higher projected public health burdens in female and low-income groups compared with male and higher-income groups; these results are consistent with a previous study in New York City (Lin et al. 2009). People with low income are more likely to live in urban areas with heat-island effects and are less likely to be able to afford air-conditioning systems or health care.

The present study has several strengths. It is one of the first studies to specifically examine respiratory morbidity and its related economic outcomes: hospitalization costs and lost productivity from days hospitalized. These outcomes may be useful metrics for public health policy makers involved in planning for potentially increased public health and economic burdens resulting from global warming in the future. Assessment of the current and future public health burden due to respiratory diseases is important because asthma (representing > 50% of the total respiratory admissions in our study population) continues to increase in the United States (Kay 2001; Redd 2002), and New York City has the highest asthma rate in the nation (Garg et al. 2003). To our knowledge, our study is one of the first to adjust projected costs for inflation and to estimate the cost of lost productivity due to hospitalization. In addition, we adjusted hospitalization costs using a cost-to-charge ratio to estimate total inpatient costs, rather than using total charges recorded at discharge, which reflect only part of the overall costs of hospitalization. Finally, we considered different climate change scenarios and used various extreme heat indicators to project effects for a range of possible future conditions.

However, our findings need to be interpreted cautiously because of uncertainties inherent in our methods. First, our projections assume that associations between extreme heat and respiratory hospital admissions would remain constant over time, which does not account for possible physiological and behavioral adaptation to extreme heat. There is currently no standard approach to model the acclimatization effect (Knowlton et al. 2007). Knowlton et al. (2007) compared estimated heat-related mortality impacts with and without acclimatization by the 2050s and concluded that the estimated increases in heat-related premature mortality would be reduced if future acclimatization were considered. In addition, our definition of lost productivity based on the length of hospital stay is an underestimate of total productivity losses, which are likely to continue after hospital discharge.

One important uncertainty is that we assumed that the size and demographic characteristics of each regional population remained constant at baseline levels in our projections of future health impacts. According to the 2010 Census, NYS had a 2.1% increase in the size of its population and a notable 19.2% increase in the size of the Hispanic population since 2000 (U.S. Census Bureau 2011b). Increases in the size of the NYS population and the proportions of vulnerable subgroups would increase the absolute number of future respiratory admissions and related economic burdens, as suggested by sensitivity analyses that indicate greater estimated impacts after accounting for projected increases in the proportions of elderly and Hispanic NYS residents.

Our projections of AT *a*) apply estimated increases in mean summer AT to the baseline, *b*) assume that the variation of the extreme heat events observed in the baseline period would remain constant in the future, and *c*) assume that the region-specific temperature thresholds estimated for the baseline period would apply in the future. Because extreme weather could become more frequent and intense (Clark et al. 2010; Dessai 2003), our projection of the future burden as a result of extreme heat events may be an underestimate. In addition, our assumption of fixed temperature thresholds does not account for potential increases in thresholds due to acclimatization. Given that it is difficult to predict the net effect of potential biases, we cannot be certain whether our projections are likely to be underestimates or overestimates.

Another uncertainty is the accuracy of our estimates of the risks of respiratory hospital admissions and the excess lengths of stay and costs related to extreme heat. To address this concern, our risk estimates were region-specific (i.e., temperature-health thresholds and risks were estimated based on each NYS weather region), and regional demographic/air pollutants and individual sociodemographics were controlled. We also used two other extreme heat indicators to validate our findings. In addition, our prediction accounted for inflation and used actual cost rather than total hospitalization charge, which overestimates cost.

A regional climate model for NYS was not available for this study, and meteorological data were projected using a global climate model that has a relatively coarse spatial resolution compared with global-to-regional climate models. However, the global climate model has been commonly used for climate projection because of the uncertainty in regional climate prediction (Dessai 2003). Another uncertainty is the selection of the CCSM3 model. This model has been used to predict future temperature and weather factors by many previous studies (Collins et al. 2006). We selected the CCSM3 model because it provides small grid coverage and information needed to project AT. This model has been shown to reliability predict observed features of current and past climates, so that it is credible for projected AT in 2046–2065 and 2080–2099 (Randall et al. 2007). In addition, sensitivity analyses suggested that estimates based on the CCSM3 global climate model are conservative compared with estimates of future climate in NYS based on the CM3 global climate model.

Uncontrolled confounding also could introduce bias. We used a two-stage Bayesian model to estimate weather–health associations for each of the 14 NYS regions that were adjusted for regional ozone levels, long-term trends, seasonal variation, weekday/weekend effects, and the 2003 Northeast blackout events. In the second stage, we pooled region-specific estimates to generate a statewide estimate adjusted for region-level characteristics, including the minimum distance to clinics for access to care, race/ethnicity, education level, poverty, and age distributions of the populations in each region.

## Conclusions

Our estimates suggest that hospital admissions for heat-related respiratory diseases in NYS in 2080–2099 will be 2–6 times higher than in 1991–2004. If other respiratory health end points (e.g., clinic visits, emergency department visits, mortality) and other heat-associated diseases were also considered, the public health and associated economic burden would be even greater. Because climate change is anticipated to increase the frequency and intensity of extreme heat events, understanding the range and scale of the current and future public health burden attributable to heat-related health effects will help policy makers develop more targeted climate-impact adaptation and mitigation strategies.
